# Separation of Fructose and Glucose via Nanofiltration in Presence of Fructooligosaccharides

**DOI:** 10.3390/membranes10100298

**Published:** 2020-10-21

**Authors:** Zulhaj Rizki, Anja E. M. Janssen, Albert van der Padt, Remko M. Boom

**Affiliations:** 1 Food Process Engineering Group, Wageningen University, P.O. Box 17, 6700 AA Wageningen, The Netherlands; anja.janssen@wur.nl (A.E.M.J.); albert.vanderpadt@wur.nl (A.v.d.P.); remko.boom@wur.nl (R.M.B.); 2 FrieslandCampina, Stationsplein 4, 3818 LE Amersfoort, The Netherlands

**Keywords:** nanofiltration, fructose glucose separation, fructooligosaccharides

## Abstract

Fructose and glucose are commonly present together in mixtures and may need to be separated. Current separation methods for these isomers are complex and costly. Nanofiltration is a cost-effective method that has been widely used for separating carbohydrates of different sizes; however, it is not commonly used for such similar molecules. Here, we report the separation of fructose and glucose in a nanofiltration system in the presence of fructooligosaccharides (FOS). Experiments were performed using a pilot-scale filtration setup using a spiral wound nanofiltration membrane with molecular weight cutoff of 1 kDa. We observed three important factors that affected the separation: (1) separation of monosaccharides only occurred in the presence of FOS and became more effective when FOS dominated the solution; (2) better separation was achieved when the monosaccharides were mainly fructose; and (3) the presence of salt improved the separation only moderately. The rejection ratio (R_f_/R_g_) in a fructose/glucose mixture is 0.92. We reported a rejection ratio of 0.69, which was observed in a mixture of 50 g/L FOS with a fructose to glucose ratio of 4.43. The separation is hypothesized to occur due to selective transport in the FOS layer, resulting in a preferential binding towards fructose.

## 1. Introduction

Fructose and glucose are sugar isomers with different properties. Fructose is sweeter than glucose [1], thus less is needed for the same sweetness. Moreover, fructose follows a different metabolic path in humans that makes it less prone to cause diabetes [2]. Therefore, fructose consumption in our diet is preferred to glucose [3]. Fructose is commonly produced by enzymatic isomerization of glucose obtained from starch [4]. After the conversion, fructose needs to be separated from the mixture, which still contains glucose.

The fact that glucose and fructose are isomers implies that they have somewhat similar chemical and physical properties [5]. Thus, they are difficult to separate by conventional means. Separation of monosaccharides has been attempted via crystallization [6], chromatography [7,8,9], and liquid membranes [10]. However, these methods are costly and difficult to operate and maintain.

Nanofiltration is a more cost-effective and flexible separation process. It has been widely used to separate or purify sugars [11,12] and oligosaccharides [13,14,15,16,17,18] from mixtures of carbohydrates. However, the separation is generally based primarily on a difference in size, and therefore cannot be used to separate sugars or isomers that have similar properties and are of similar size [19,20,21].

Nanofiltration is already used for the purification of fructooligosaccharides (FOS) to remove small sugars from long-chain oligosaccharides [13,18,22]. FOS, in its native form, is a mixture of oligofructoses with various degrees of polymerization (DP). The DP determines the health benefits and functional properties of the FOS products [23,24]. Monosaccharides add to the caloric content of the mixture and add sweetness, which is not always desired.

**Figure 1:**
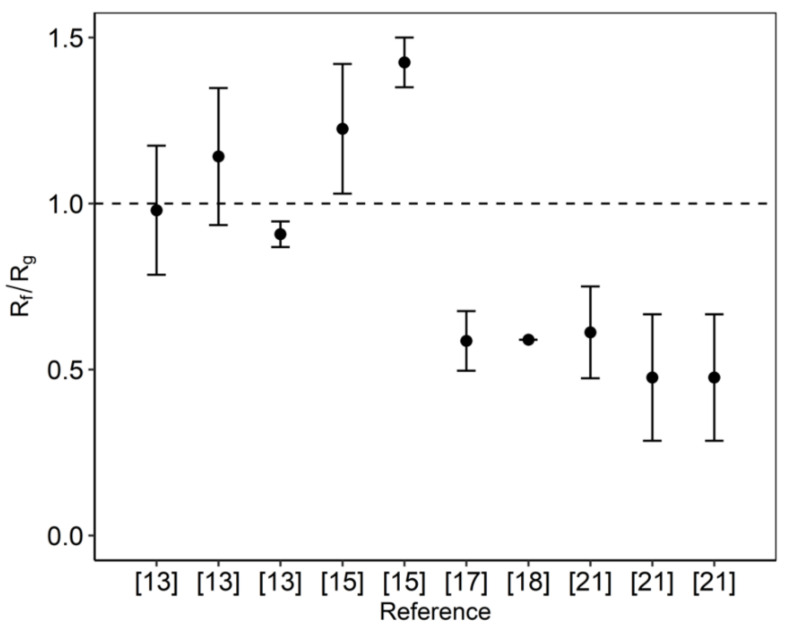
Rejection ratio of fructose over glucose (R_f_/R_g_) from various references [13,15,17,18,21] under different setups and feeds.

FOS purification using nanofiltration aims to achieve a higher content of long oligosaccharides in the product. Separation of the monosaccharides was not expected and therefore often ignored or not reported. Based on the similar molecular sizes of glucose and fructose, it was expected that both molecules would be rejected more or less equally. However, this was not observed. In some studies, the rejection of fructose was much lower than that of glucose [17,18,21]; in other studies the opposite was observed [13,15]. The ratio of the rejection of fructose and glucose from these studies are shown in Figure 1. Various types of membranes and feed sources were used that may relate to their difference in selectivity. Unfortunately, to date, this phenomenon has not been explored and explained. We therefore investigate this separation of monosaccharides and the influence of the presence of larger sugars on this separation.

Both glucose and fructose show polymorphic behaviour in solution; several configurations of the sugars are present simultaneously. Aside from their open chain form, glucose has the pyranose form, whereas fructose has both pyranose and the furanose forms [25,26]. Since it has fewer carbon atoms in its main ring, the furanose form is smaller than the pyranose form. In equilibrium, around 25% of fructose is in its furanose form, which makes its average size smaller than that of glucose [27]. Therefore, less rejection is often observed for fructose compared with glucose.

Both glucose and fructose were reported to have affinity with cations, such as sodium and calcium. This is used in affinity separations such as chromatography [7]. Both sugars show different affinity towards specific ions, and therefore, one sugar migrates faster than the other in a chromatographic column containing a specific sequestered ion. If the ion is not sequestered to the column, a sugar-ion complex that is larger than a free sugar is formed [28,29,30]. In addition, this complexation transforms a neutral sugar into a charged complex, which may also affect the sieving mechanism in nanofiltration. Separating sugars with the aid of cations was previously reported with a cellulose acetate membrane [31]; residual trace cations in the industrial FOS mixture may have been responsible for this.

In this research, we study the separation of fructose and glucose using nanofiltration in the presence of FOS. The feed composition was varied to find the key factor for this separation and its mechanism. Further development of this separation will be useful for finding alternative methods to separate sugar isomers, which currently requires more costly processes.

## 2. Materials and Methods

### 2.1. Materials

This work is inspired by the findings in previous works related to the nanofiltration of FOS [17,32]. Therefore, a similar FOS is used in this research. In addition, fructose and glucose were added to the FOS solution in various concentrations. The experiments in this research were performed using mixtures of FOS syrup (Frutalose L85), glucose (d-(+)-glucose monohydrate), and fructose (d-(−)-fructose) in various compositions. The Frutalose syrup was kindly provided by Sensus (Roosendaal, the Netherlands) and the monosaccharides were purchased from Merck KGaA (Darmstadt, Germany). The FOS syrup contained 75 wt % of dry matter, consisting of carbohydrates with a degree of polymerization (DP) ranging from 1 to 10. Monosaccharides were added to this mixture, taking into account the monosaccharides that were already present in the FOS syrup. The carbohydrate composition (dry basis) of the FOS syrup is presented in Table 1. In this table, the oligosaccharides with DP5 and higher are shown as a single lumped component.

Salts were added to the mixtures in the form of NaCl (purity ≥99.5%) and CaCl_2_ (purity ≥93%). Both salts were purchased from Sigma-Aldrich (Merck KGaA, Darmstadt, Germany).

### 2.2. Filtration Experiment Setup

All experiments were performed using a pilot-scale membrane unit with a total process volume of 7.5 L, which included a volume of 2.5 L inside the equipment [16,17,21,32]. The unit was connected with flow sensors at both permeate and retentate streams, a pressure control, a heat exchanger, and refractive index sensors at the outlet streams. A commercial spiral wound polyamide nanofiltration membrane (GE type, 1812 model) from General Electric (GE Osmonics, Sterlitech, Kent, WA, USA) was used. The membrane has a molecular weight cut off (MWCO) of 1 kDa (tested with polyethylene glycol). Before the main experiments, new membranes were treated under pressure (4 bar) with demi water for 2 h to remove the trace of additives. This procedure was suggested by the membrane supplier.

The experiments were carried out at a fixed temperature of 45 °C, a transmembrane pressure (TMP) of 16 bar, and a cross-flow velocity of 0.10 m/s. The membrane water flux at this operating condition was reported as 8.24 ± 0.59 × 10^−6^ m^3^/(s. m^2^) [32].

Each experiment was operated until a steady state condition was achieved. This was indicated by stable refractive indices in both permeate and retentate streams. After steady state conditions were achieved, samples were taken from the feed and permeate and retentate streams to be analysed for their carbohydrate composition.

We carried out filtration experiments with various concentrations and compositions of carbohydrates, with and without addition of salts. Mixtures of carbohydrates were prepared by combining the FOS syrup with glucose and fructose powder and diluting with demineralized water. All experiments that involved FOS were prepared by diluting to 5% of the FOS syrup resulting in 35 g/L total oligosaccharides (DP ≥ 3). Except the experiments that investigated the effects of the ratio of mono- to oligosaccharides, all experiments had the same overall monosaccharide concentration of 16 g/L but with various ratios of fructose to glucose. In the experiments that involved salts, the salts were added to have a cation concentration of 0.25–2 g/L. The experiments performed in this study are summarized in Table 2.

### 2.3. Analyses

The carbohydrate concentrations of all samples were analysed using high-performance liquid chromatography on a Shodex column (KS-802 8.0 × 300 mm) that was integrated with a refractive index detector (Shodex RI-501, Showa Denko, Japan). The separation in the column was carried out at 50 °C with demineralized water (Milli-Q, Merck, Darmstadt, Germany) as eluent at a flow rate of 1 mL/min. Aside from the monosaccharides, the other carbohydrates were quantified in regard to their DPs. Oligosaccharides with DP5 or higher were quantified as one grouped component. The monosaccharides, on the other hand, were quantified as fructose and glucose separately.

Carbohydrate analysis was done for both permeate and retentate streams for every experiment. The rejection coefficient for each sugar was then calculated using Equation (1).

R_i_ = 1 − C_p,i_/C_r,i_, (1)

with i = fructose (f) or glucose (g).

## 3. Results and Discussion

This work was inspired by a finding while studying the separation of FOS with nanofiltration, with the composition shown in Table 1. We use this solution as a benchmark, because it gave a surprising selectivity between fructose and glucose during nanofiltration. The selectivity was presented as a rejection ratio, R_f_/R_g_. Its value during nanofiltration of the FOS solution was found to be 0.59, and this is presented as a dot-dashed line in the figures. We compared this solution with a mixture containing the same concentrations of fructose and glucose without the oligosaccharides, which did not show good separation, with R_f_/R_g_ = 0.92. This value is presented as a dotted line. We found three essential differences in the FOS solution that may cause the separation: (1) oligosaccharides were present; (2) fructose and glucose were present in non-equimolar concentrations; (3) as a natural mixture, the FOS solution may contain some trace ions. We investigate these three factors in the following sections. The rejection of both sugars as well as their rejection ratios are presented in the following section.

### 3.1. The Effect of Oligosaccharides

**Figure 2:**
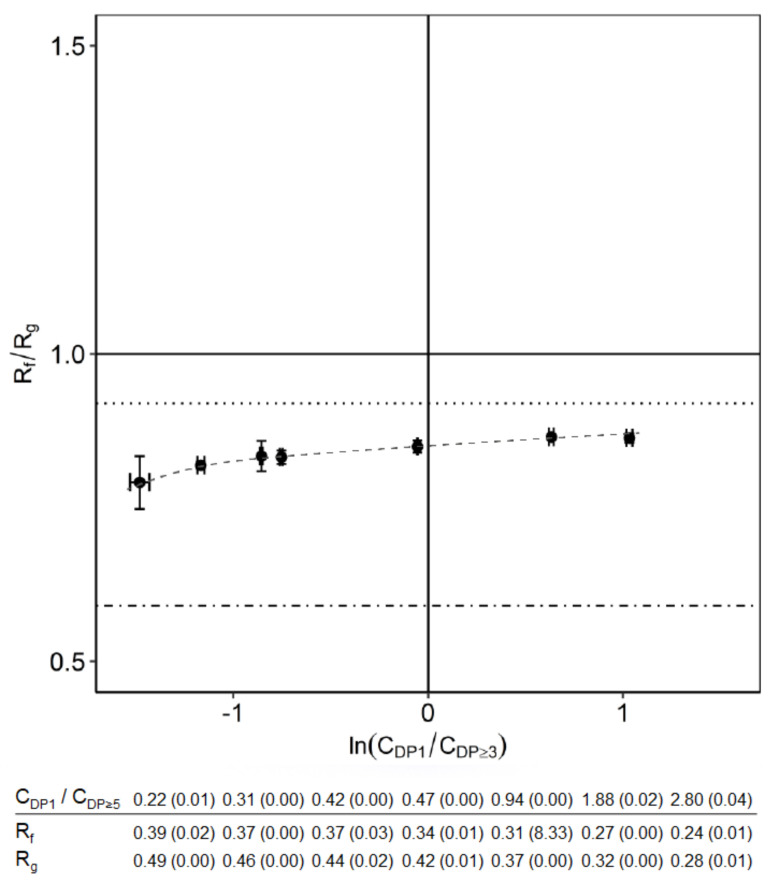
Effect of oligosaccharides concentration on selectivity in a nanofiltration system. The dotted line is the reference rejection ratio for a mixture with only fructose and glucose. The dot-dashed line is the reference rejection ratio for the FOS mixture. The dashed line is shown to guide the eye. The 95% confidence interval for concentration ratio and the rejections are shown inside the brackets.

We varied the ratio of mono- to oligosaccharides in the FOS mixture by adding fructose and glucose. The oligosaccharides concentration was kept constant at 35 g/L. The total monosaccharides concentration was varied (Table 2, experiment A) while keeping the final concentration of fructose similar to that of glucose. The effect on their selectivity, R_f_/R_g_, is shown in Figure 2. The horizontal axis in Figure 2 can be divided into two zones: the positive side of the axis where the monosaccharides are dominant; and the negative axis where the oligosaccharides are dominant. Extending this axis towards infinity represents systems with only monosaccharides (right-hand side) and only oligosaccharides (left-hand side).

A selectivity <1 represents higher retention of glucose. This selectivity is independent of the ratio of mono- to oligosaccharides, with higher concentrations of monosaccharides but tending towards lower values with lower ratios indicating a better separation between fructose and glucose if the oligosaccharide concentration is high. All results were between the two references values (indicated by the dotted line and dot-dashed line; see previous section). This implies that the presence of oligosaccharides (or other dissolved and retained components) indeed influences and promotes the separation of fructose and glucose. The FOS reference naturally has an excess of oligosaccharides compared with the monosaccharides, with ln(C_DP1_/C_DP ≥ 3_) = −2.2, which positions further left (Figure 2). The result shows a tendency towards this reference value.

### 3.2. Effect of Monosaccharides Composition

**Figure 3:**
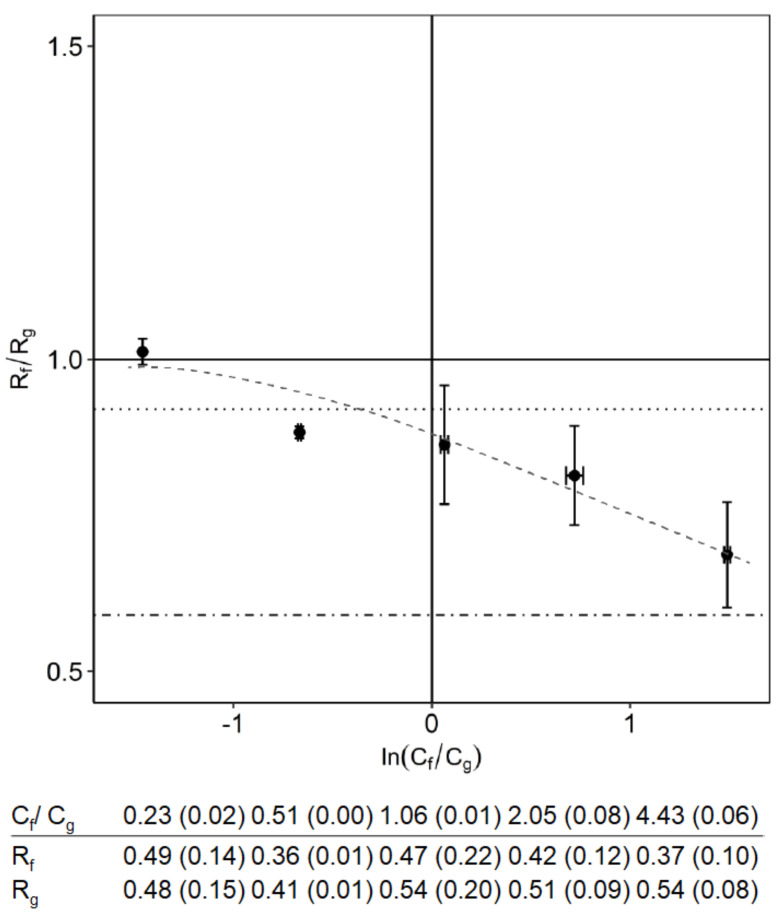
Effect of the monosaccharide composition on selectivity in a nanofiltration system. The total oligosaccharide concentration was 35 g/L, and the total monosaccharide concentration was 16 g/L. The dotted line is the reference rejection ratio for a mixture with only fructose and glucose. The dot-dashed line is the reference rejection ratio for the FOS mixture. The dashed line is shown to guide the eye. The 95% confidence interval for concentration ratio and the rejections are shown inside the brackets.

Keeping the oligosaccharide and total monosaccharide concentrations constant (Table 2, experiment B), we varied the ratio between fructose and glucose and show the results in Figure 3. The separation was enhanced with an excess of fructose. This was mainly due to a lower retention of fructose, whereas the retention of glucose did not really change. With an excess of glucose, the separation factors tended to 1, which is even higher than without any FOS present.

**Figure 4:**
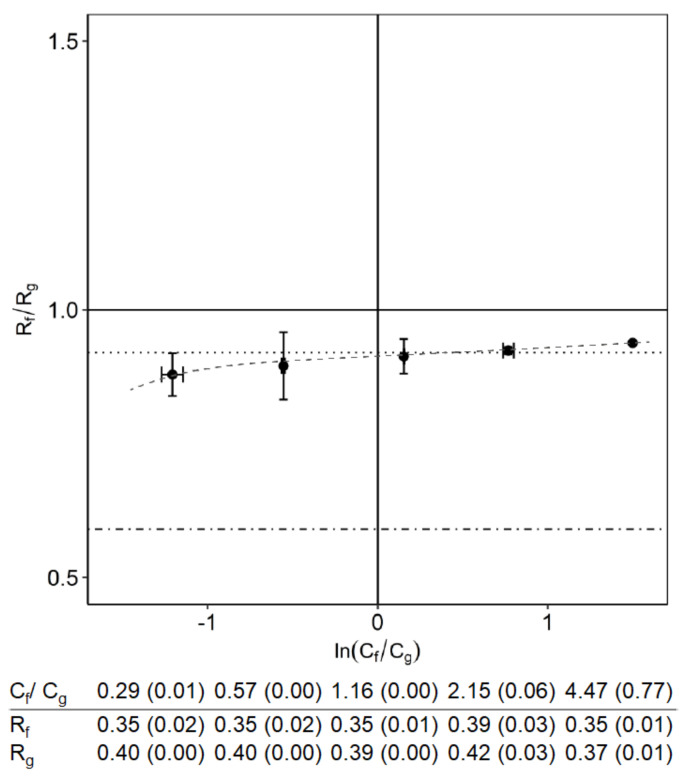
The rejection ratio of fructose and glucose in a nanofiltration system with only monosaccharides. The total monosaccharide concentration was 16 g/L. The dotted line is the reference rejection ratio for a mixture with only fructose and glucose. The dot-dashed line is the reference rejection ratio for FOS mixture. The dashed line is shown to guide the eye. The 95% confidence interval for concentration ratio and the rejections are shown inside the brackets.

The better separation with an excess of fructose was only observed when FOS was present. When the oligosaccharides were absent, the rejection ratios of fructose and glucose were almost constant at various ratios of fructose to glucose (Figure 4). The reason that this value is slightly below 1 is because fructose is partly in the furanose form, which is slightly smaller than fructose in the pyranose form.

### 3.3. Effect of Electrolytes

**Figure 5:**
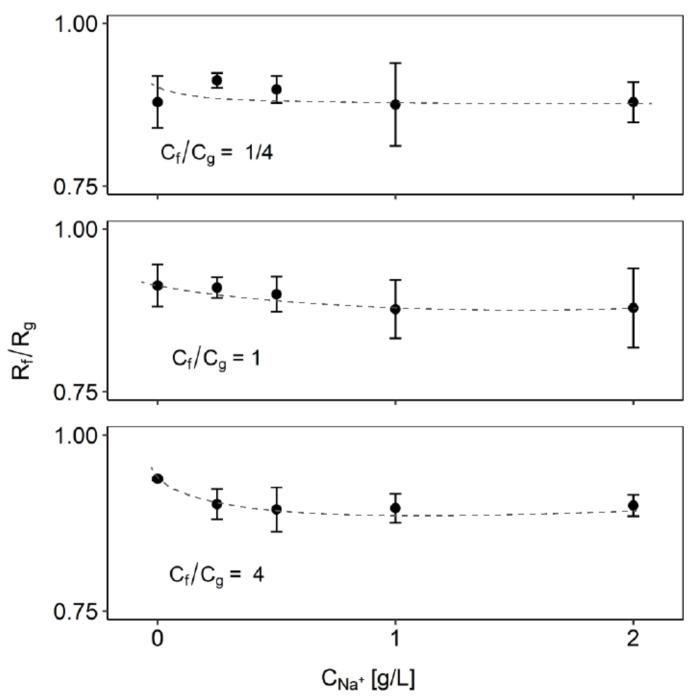
Effect of addition of salt on the separation of fructose and glucose in nanofiltration with only monosaccharides. The total monosaccharide concentration was 16 g/L. The dashed lines are shown to guide the eye.

The addition of small amounts of salts increased the separation somewhat, as indicated by a decrease in the rejection ratio of fructose and glucose. However, at some point, adding more salts did not change the separation further (Figure 5). Both sugars interact with the cations, albeit at different levels. Depending on the ion, one sugar may have a stronger affinity than the others. In a mixture with only monosaccharides, the addition of NaCl improved the separation by lowering the rejection ratio. This implied that the retention of glucose increased more strongly than that of fructose, based on the stronger interaction of glucose with sodium ions. At some point, the addition of salt did not improve the separation any further. We saw again that the effect was mostly present with an excess of fructose; with equimolar concentrations or an excess of glucose, we saw little influence of the metal ions.

**Figure 6:**
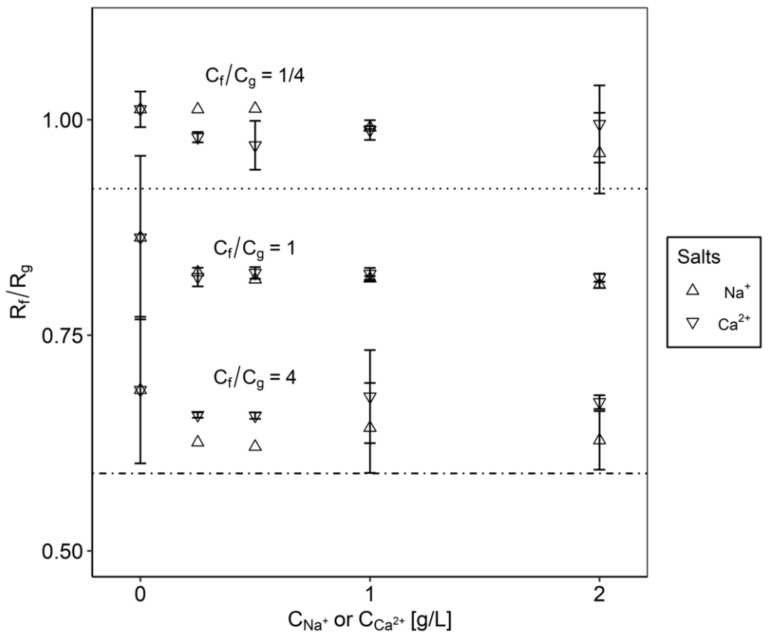
Effects of the addition of salt on the separation of fructose and glucose in the FOS system. The oligosaccharide concentration was 35 g/L, and the monosaccharide concentration was 16 g/L.

In solutions with FOS (Figure 6), we observed similar trends, but with much larger selectivity. An equimolar concentration of glucose and fructose gave a slight increase in the separation (reduction in the selectivity factor), but only with small amounts of salt. Both sodium and calcium affected the separation similarly. With an excess of glucose present (*C*_f_/*C*_g_ = 1/4), there was no significant effect of either Ca or Na on the separation. However, with an excess of fructose, the separation was larger (lower separation factor), and Na had a stronger effect than Ca (*C*_f_/*C*_g_ = 4).

### 3.4. General Discussion

Separation between fructose and glucose during nanofiltration only occurred at a significant level in the presence of oligosaccharides, and this separation was stronger with low concentrations of monosaccharides relative to the oligosaccharides. The separation was much more pronounced with an excess of fructose relative to glucose. There was some influence of the presence of small concentrations of metal ions (Ca or Na), but the addition of salt had a much smaller effect than the composition of the monosaccharides.

We expect that a concentration polarization layer is present near the membrane. In this layer, the FOS concentration gradually increases from the bulk to the surface of the membrane. This polarization layer acts as an additional barrier for the transport of components towards the permeate. We expect that the selectivity between the monosaccharides is due to difference in transport in the polarization layer.

This sugar transport across the polarization layer can be affected by the interaction between FOS and the monosaccharides, e.g., by hydrogen bonding. FOS molecules contain free hydroxyl groups that can form hydrogen bonds. Additionally, fructose and glucose have free hydroxyl groups that allow them to form hydrogen bonds with FOS. Topographically, the hydroxyl groups in fructose were positioned differently, compared with glucose. Fructose was reported to form stronger hydrogen bonds [33,34]. Therefore, fructose may have more affinity towards FOS than glucose. However, these fructose molecules will take up space between the FOS molecules, which will exclude the glucose molecules, leading to a net selectivity.

The necessary presence of the polarization layer explains the results described in Section 3.1 and Section 3.2 Without FOS in the polarization layer, the only selectivity is through sieving by the membrane. As the difference in the size of the monosaccharides is small, a low and constant selectivity was observed. As soon as FOS are present in the solution, selective transport through the polarization layer results in lower fructose rejection. When the concentration of oligosaccharides is low, the polarization layer features low concentrations. Therefore, there may still be some residual selectivity. With higher concentrations of FOS in the mixture, the polarization layer is more concentrated, the selective transport is more effective, and the separation is enhanced.

We therefore hypothesize that the selective transport occurs due to hydrogen bonding between mono- and oligosaccharides, leading to preferential binding of fructose, and thus, exclusion of glucose from the concentration polarization layer. Therefore, the presence of salts does not significantly affect this mechanism. The small effects that were observed with ions may have been because of weak complexation, but may also have been because of different ionic strengths. However, these effects were small.

This research uses a nanofiltration membrane with MWCO of 1 kDa. Considering the purpose of this study, separating fructose and glucose, the idea of using smaller membranes may arise. However, this study shows that such separation only occurs in the presence of FOS. A complete rejection of FOS may occur using tighter membranes. This will increase the resistance, hence lower the flux. In addition to that, the FOS increases the solution viscosity, which synergistically decreases the flux.

## 4. Conclusions

Separation of fructose and glucose was observed using nanofiltration in the presence of FOS. The FOS formed a polarization layer on the membrane surface, which acted as a selective barrier for the monosaccharides. Fructose had a stronger interaction with FOS, therefore glucose was excluded from the concentration polarization layer; hence, fructose permeates faster than glucose. This separation occurs only when the FOS is present at a sufficient concentration relative to the monosaccharides.

The separation is strongest with an excess of fructose relative to glucose. The presence of low concentrations of metal ions (sodium or calcium) enhanced the selectivity somewhat, but higher concentrations had no additional effect. This may be due to charge interaction with the membrane itself. We did not observe any strong indication of complexation between monosaccharides and metal ions.

## Figures and Tables

**Table 1:** Carbohydrate composition (on a dry weight basis) of fructooligosaccharides (FOS) syrup used in this research.

Component	Concentration (wt %) ^a^
Glucose	6.9 ± 0.5
Fructose	2.0 ± 0.2
DP2	10.0 ± 1.1
DP3	23.6 ± 1.5
DP4	23.9 ± 1.4
DP5 and higher	33.6 ± 2.3

^a^ Uncertainties were calculated based on a 95% confidence interval.

**Table 2:** Variation of carbohydrates and salts concentration in all experiments.

Experiments	C_DP1_	C_DP ≥ 3_	C_DP1_/C_DP ≥ 1_	C_fru_/C_glu_	C_Na_^+^ or C_Ca_^2+^
	(g/L)	(g/L)			(g/L)
Experiment A	9–140	35	0.25–4	1	–
Experiment B	16	35	0.45	0.25–4	–
Experiment C	16	–	–	0.25–4	–
Experiment D	16	35	– and 0.45	0.25–4	0.25–2
